# Oncolytic efficacy of thymidine kinase-deleted vaccinia virus strain Guang9

**DOI:** 10.18632/oncotarget.17125

**Published:** 2017-04-15

**Authors:** Lili Deng, Jun Fan, Yuedi Ding, Jue Zhang, Bin Zhou, Yi Zhang, Biao Huang

**Affiliations:** ^1^ Key Laboratory of Nuclear Medicine, Ministry of Health, Jiangsu Key Laboratory of Molecular Nuclear Medicine, Jiangsu Institute of Nuclear Medicine, Wuxi, Jiangsu, China

**Keywords:** vaccinia virus, oncolytic virotherapy, Tian Tan strain Guang9, cancer therapy, thymidine kinase

## Abstract

Oncolytic virotherapy is being developed as a promising platform for cancer therapy due to its ability to lyse cancer cells in a tumor-specific manner. Vaccinia virus has been used as a live vaccine in the smallpox eradication program and now is being potential in cancer therapy with a great safety profile. Vaccinia strain Guang9 (VG9) is an attenuated Chinese vaccinia virus and its oncolytic efficacy has been evaluated in our previous study. To improve the tumor selectivity and oncolytic efficacy, we here developed a thymidine kinase (TK)-deleted vaccinia virus based on Guang9 strain. The viral replication, marker gene expression and cytotoxicity in various cell lines were evaluated; antitumor effects *in vivo* were assessed in multiple tumor models. *In vitro*, the TK-deleted vaccinia virus replicated rapidly, but the cytotoxicity varied in different cell lines. It was notably attenuated in normal cells and resting cells *in vitro*, while tumor-selectively replicated *in vivo*. Significant antitumor effects were observed both in murine melanoma tumor model and human hepatoma tumor model. It significantly inhibited the growth of subcutaneously implanted tumors and prolonged the survival of tumor-bearing mice. Collectively, TK-deleted vaccinia strain Guang9 is a promising constructive virus vector for tumor-directed gene therapy and will be a potential therapeutic strategy in cancer treatment.

## INTRODUCTION

Oncolytic virotherapy has recently been emerged as a promising and appealing strategy for combating cancer [1–5]. Replicating viruses infected to tumor cells will lead to an expanding replication within the tumor, then lysing tumor cells and spreading within tumor tissues. Currently, a variety of viruses are under investigation for treatment of cancer, including adenovirus, herpes virus, Newcastle disease virus, and vaccinia virus [6–9]. Of these, vaccinia virus has some excellent characteristics over other viruses, such as large transgene-encoding capacity, efficient foreign gene expression using its own enzyme systems, and selective replication in tumor tissues.

Vaccinia virus has been used as a live vaccine in the smallpox eradication campaign and recently as a vaccine against other infectious diseases and cancer [10, 11]. Its safety profile has been established through the use as a smallpox vaccine in tens of millions of humans globally. However, due to the safety as a systemically administered replicating virus, it has not been extended applied as the tumor-directed gene therapy vector [12, 13]. Case reports of vaccinia-associated encephalitis have been described in the immunosuppressed population [14–17]. Therefore, various modifications toward improving tumor specificity and safety have been explored [18–20].

In the previous study, pathogenicity was decreased significantly in viral thymidine kinase (TK)-negative phenotype [21]. It was also demonstrated that the disruption of the vaccinia virus TK gene led to significant attenuation in normal tissues, while tumor tissues were able to complement this gene deletion and support viral replication [22, 23]. A recent research showed that a systemically injection of TK-deleted vaccinia virus expressing luciferase induced about 3000-fold higher gene expression in tumors compared with all other normal tissues [24]; other studies also indicated that viral TK deletion resulted in specific replication in tumor tissues [23, 25]. The viral TK gene is not required for dividing cells, while is essential for infection of resting cells [21], permitting tumor-selective replication *in vivo*. All of these indicate that tumor targeting can be enhanced through viral thymidine kinase gene deletion.

Such oncolytic vaccinia virus (TK-deleted virus) has demonstrated promising results in the treatment of cancer in Western Reserve strain (WR) [23, 24, 26, 27] and Lister strain [28]; however, the potential as tumor-selective agent of Chinese vaccine strain with TK deletion remains unknown. Vaccinia strain Guang9 (VG9) is an attenuated Chinese vaccinia virus, which was derived from Chinese vaccinia strain Tian Tan (VTT) by consecutive plaque-cloning selection [29–31]. The biological characteristics of VG9 have been studied clearly and it is supposed to become an encouraging construction of recombinant vaccinia virus vector [32]. We have previously demonstrated the antitumor effect of VG9 both *in vitro* and *in vivo*. In this study, we constructed a TK-deleted vaccinia virus expressing enhanced green fluorescent protein (EGFP) based on VG9 strain. We examined the antitumor effects of this recombinant vaccinia virus both *in vitro* and *in vivo* and compared the results with TK-deleted WR strain.

## RESULTS

### Construction of a TK-deleted vaccinia virus

**Figure 1:**
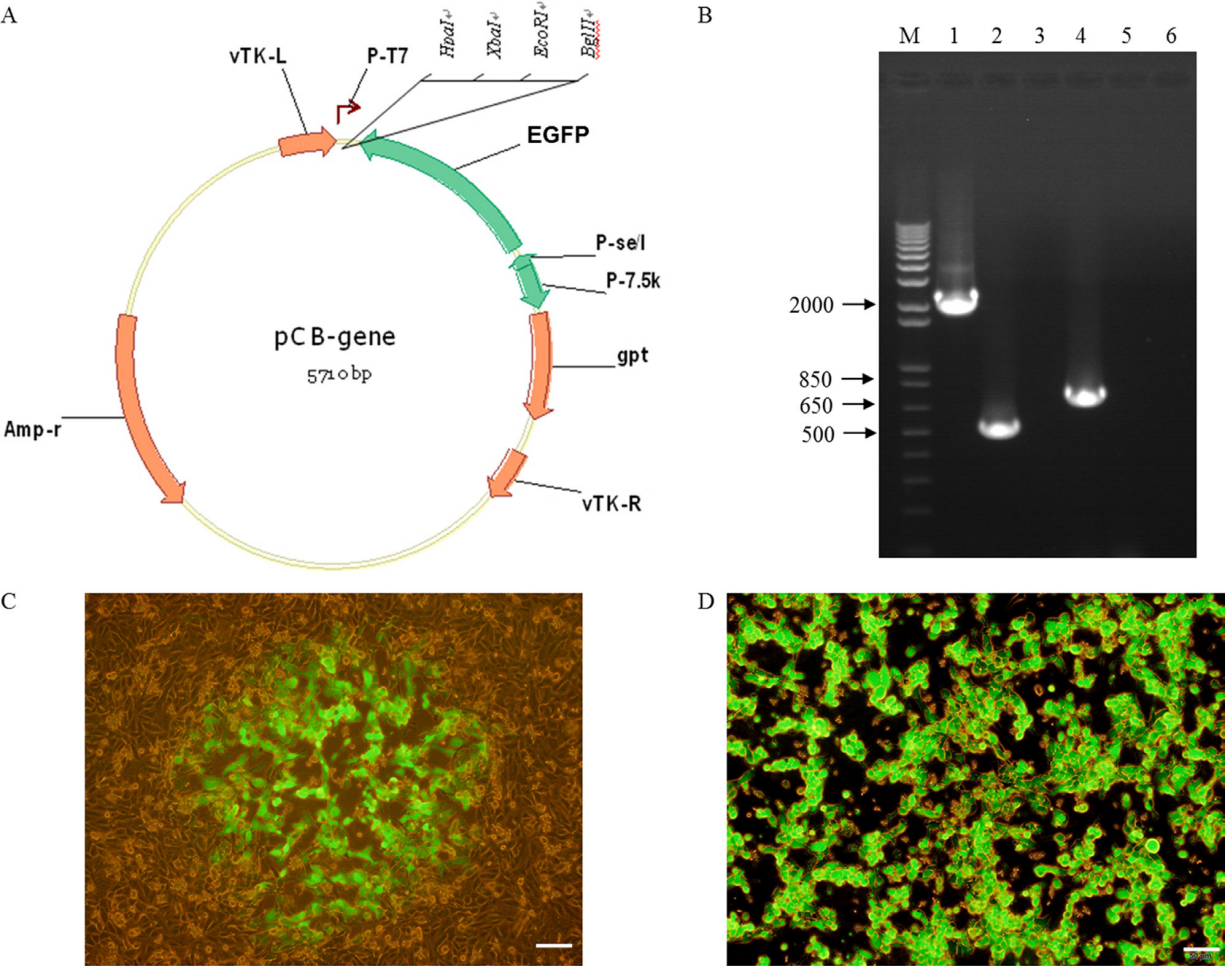
Construction of a TK-deleted vaccinia virus (**A**) Generation of a shuttle plasmid containing EGFP gene. (**B**) PCR analysis of viral DNA from VG9-EGFP confirming homologous recombination. M, 1Kb Plus DNA Ladder (Invitrogen); Lane 1 (VG9-EGFP), P1/P2 primers amplify a 2210-bp fragment across the region of recombination, which confirms the absence of TK. The TK positive fragment (534-bp) amplified of VG9 is shown in Lane 2 and negative control (H_2_O) is shown in Lane 3. Lane 4 (VG9-EGFP), P3/P4 primers amplify EGFP to product a 736-bp band, which is absent in VG9 (Lane 5). Negative control (H_2_O) is shown in Lane 6. (**C**) Recombinants expressed green fluorescent protein. (**D**) Vero cells infected with VG9-EGFP for 2 days. Bar: 50 μm.

The TK-deleted recombinant vaccinia virus expressing EGFP gene was generated from the attenuated Chinese vaccinia strain VG9. A shuttle plasmid was used to insert EGFP into TK locus by homologous recombination, creating TK-deleted vaccinia virus, VG9-EGFP (Figure 1A). PCR using primers designed to amplify the EGFP gene and spanning the site of recombination confirmed the insertion of EGFP (Figure 1B). Fluorescent microscope also demonstrated that recombinants produced green fluorescent protein (Figure 1C). At 2 days post-infection of VG9-EGFP, all plaques were observed green fluorescent expression (Figure 1D).

### Virus replication *in vitro*

**Figure 2:**
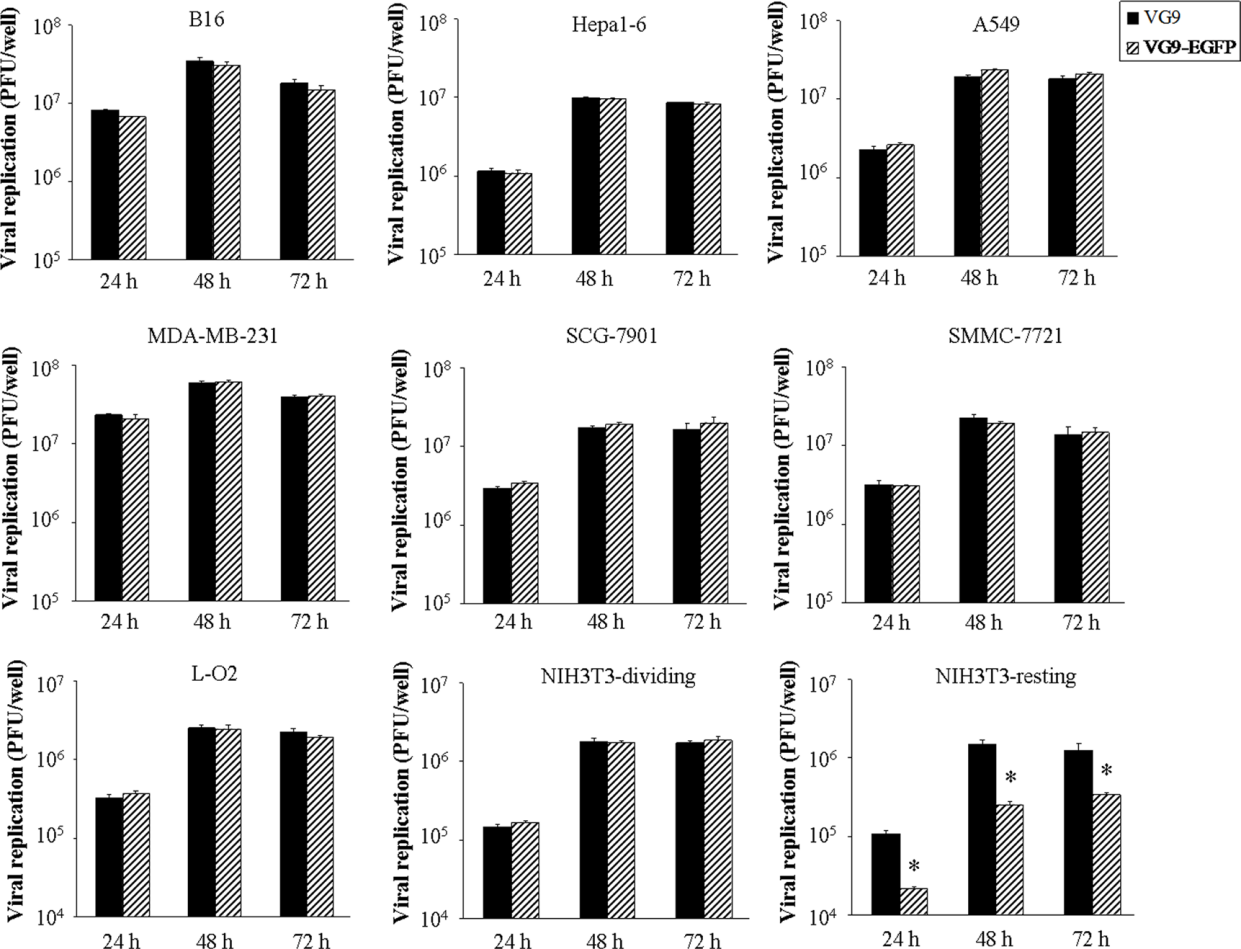
Viral replication *in vitro* Various cancer cell lines (B16, Hepa1-6, A594, MDA-MB-231, SCG-7901, SMMC-7721) and two normal cell lines (L-O2, NIH3T3) in 12-well plate were infected with VG9 or VG9-EGFP at 0.1 MOI and samples were collected at indicated times. Virus titers were determined on BSC-40 cells. Each bar represents the mean ± SD (*n* = 3). *p < 0.05 versus VG9 group.

The ability of TK-deleted vaccinia strain VG9-EGFP to replicate and spread was determined in various cancer cell lines and normal cell lines. The yield of infectious virus in cells at indicated time points was quantified by plaque assays in BSC-40 cells. As shown in Figure 2, VG9-EGFP increased rapidly in all types of cancer cells, reaching to a maximum within 48 hours with no significant difference compared with wild-type (VG9). Maximum virus production occurred in MDA-MB-231 cells, followed by B16 cells, while the yield in Hepa1-6 cells was a little lower. The viral yield of VG9-EGFP was much less in normal cells compared with cancer cells. These results indicated that TK-deleted vaccinia strain has gained specificity to cancer cells while the ability of replication is as well as wild-type. However, the VG9-EGFP production in confluent NIH3T3 cells was notably lower compared with wild-type virus (VG9) at all indicated time points. The results were consistent with the supposition that TK deletion would lead to attenuation in resting cells, while similar replication to wild-type in dividing cells owing to sufficient nucleotides provided for synthesis.

### Green fluorescent protein expression

**Figure 3:**
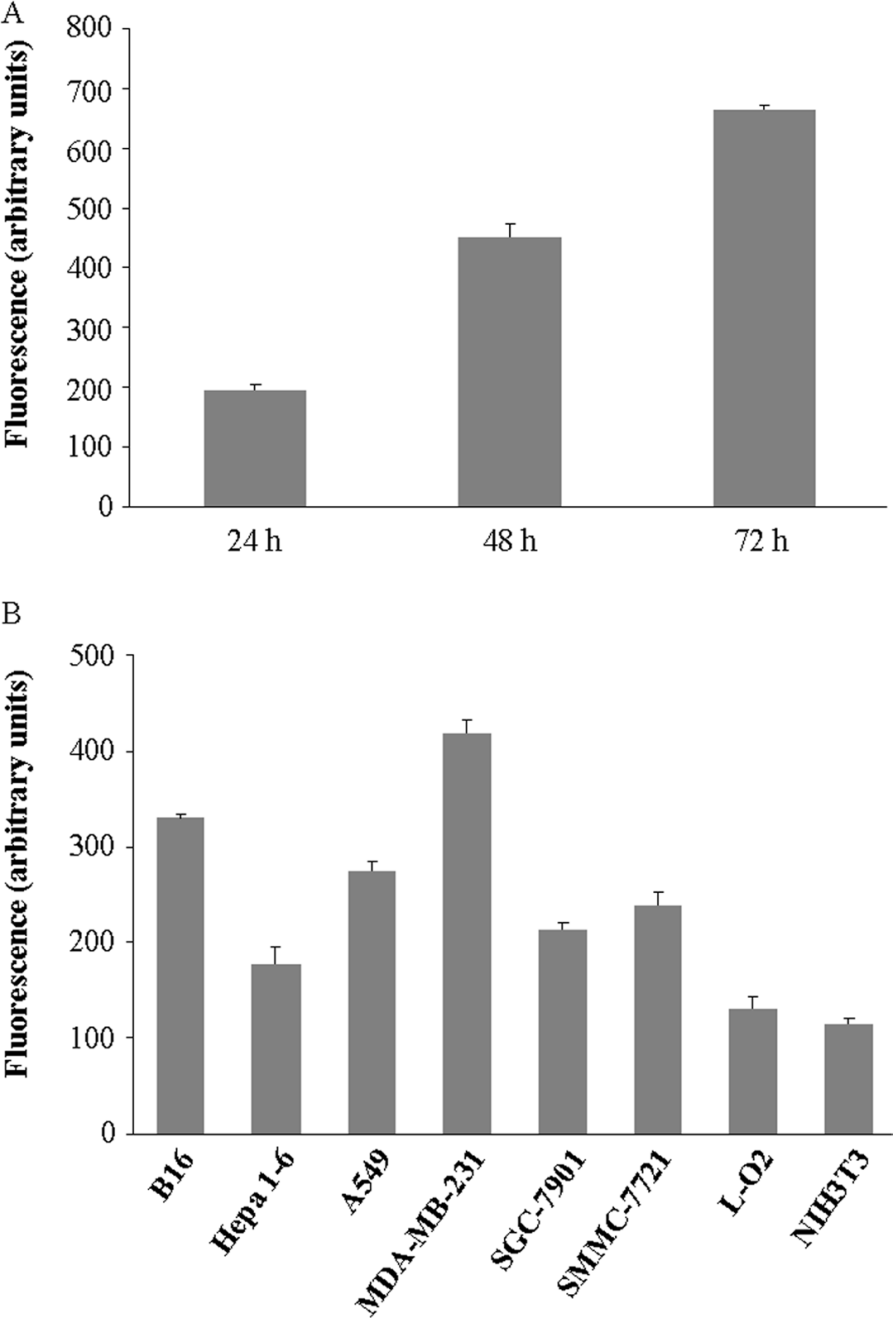
EGFP expression in different cell lines (**A**) Total fluorescence of VG9-EGFP from Vero cells at indicated time points. (**B**) Total fluorescence of VG9-EGFP from various cancer cell lines and normal cell lines harvested at 72 hour time point. Each bar represents the mean ± SD (*n* = 3).

In order to quantify the EGFP expression, total fluorescence of VG9-EGFP from Vero cells was determined at various time points. As shown in Figure 3A, the intensity of fluorescent was increased with time going on, indicating continuous expression of EGFP. Then, we observed whether the fluorescence was different in various cancer cell lines and normal cells (Figure 3B). The results showed that the fluorescence produced by MDA-MB-231 cells was most intensive, followed by B16 cells, while the fluorescence intensities in two normal cell lines were much lower, which were consisted with the ability of viral replication.

### Cytotoxic effect *in vitro*

**Figure 4:**
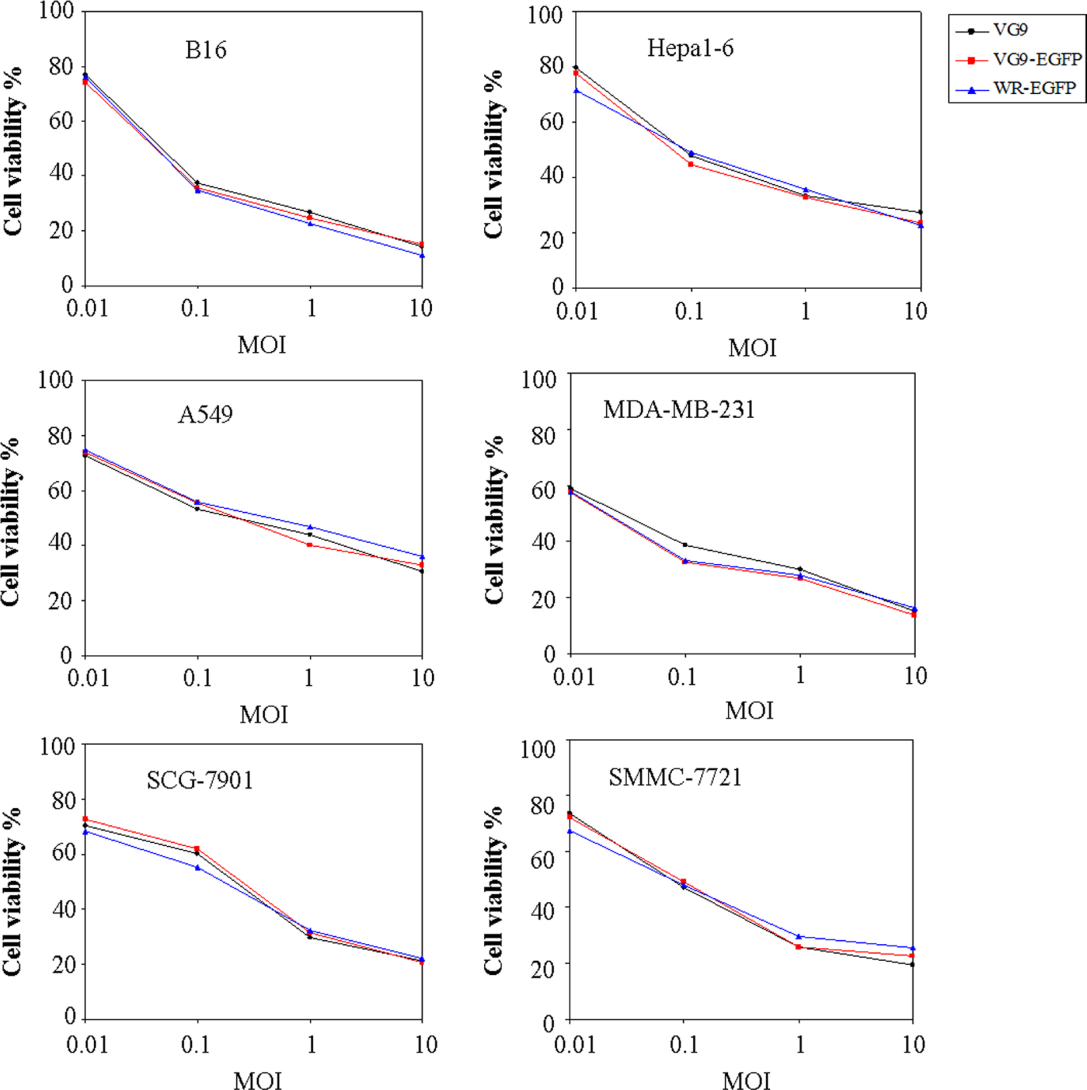
*In vitro* cytotoxicity assay B16, Hepa1-6, A594, MDA-MB-231, SCG-7901, and SMMC-7721 cells were infected with VG9, VG9-EGFP, or WR-EGFP at different MOIs. 72 hours post-infection, cell viability was measured by MTT assay.

We then evaluated the oncolytic potency in various cancer cell lines. Cells were cultured in 96-well plates and then infected with increasing doses of viruses. The cell viability was measured by MTT assay at 72 hours after infection (Figure 4). The results showed that the cytotoxic effects of VG9-EGFP were similar to VG9 in all cell lines. MDA-MB-231 and B16 cells were more sensitive to virally-induced cell killing with the survival of less than 20% at 10 MOI. The cytotoxic effects between TK-deleted VG9 strain and WR strain had no significant difference. Results indicated that TK-deleted vaccinia strain remained its oncolytic ability with diverse sensitivity to different tumor cell lines.

### Virus replication *in vivo*

The *in vivo* replication of TK-deleted vaccinia strain was assessed 5 days post-infection by analyzing virus titers in tumors and normal organ tissues (brain, spleen, lung, liver, and kidney). Infectious viruses in these tissues were titered on BSC-40 cells, and viral yield was calculated per milligram tissue (Table 1). Virus administration directly into the peritoneal cavity led to markedly higher viral yield in tumors compared with other normal organ tissues.

**Table 1:** Viral yield in tumor and normal tissues^a^

	VG9	VG9-EGFP
Tumor	1.32 (1.06–1.82) × 10^5^	9.6 (7.86–16.24) × 10^4^
Brain	26 (0–102)	8 (0–16)
Lung	2 (0–18)	0
Liver	0 (0–8)	2 (0-6)
Spleen	86 (15–264)	11 (0–92)
Kidney	36 (27–76)	24 (0–65)

^a^The median (range) viral yields, PFU/mg tissue on day 5 after injection with VG9 or VG9-EGFP.

### Antitumor effect in multiple tumor models

**Figure 5:**
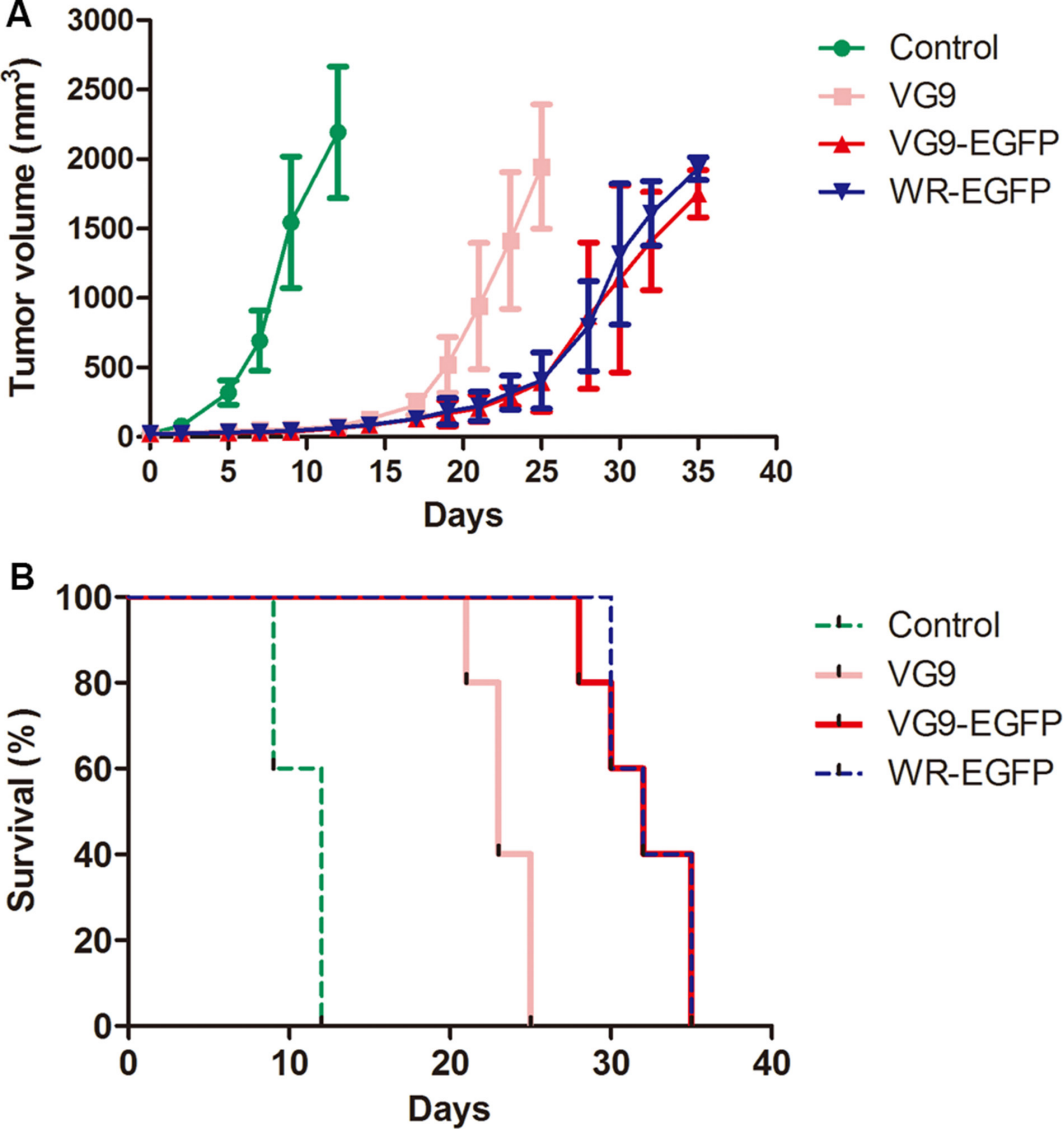
Antitumor efficacy in a murine melanoma model (**A**) Tumor development in mice treated with PBS (Control group), VG9 (VG9 group), or TK-deleted vaccinia virus (VG9-EGFP group, WR-EGFP group). (**B**) Kaplan-Meier survival curves for B16 tumor-bearing mice treated with PBS (Control group), VG9 (VG9 group), or TK-deleted vaccinia virus (VG9-EGFP group, WR-EGFP group). *n* = 5 per group.

The oncolytic efficacy of TK-deleted vaccinia strain was evaluated in two tumor models. In mouse cancer model, immunocompetent C57BL/6 mice bearing B16 murine melanoma tumors were injected intratumorally with PBS (control), VG9 or TK-deleted vaccinia virus when tumors reached 3~5 mm in diameter, and followed for survival. As shown in Figure 5A, tumors in control group had notably increased in size by 12 days following the initiation of treatment, while those in virus-treated groups had stabilized. However, by 25 days post-treatment, an increase in tumor burden was observed in VG9-treated mice; in contrast, tumor development was delayed in the TK-deleted groups (VG9-EGFP and WR-EGFP). All control mice died within 12 days, while VG9-treated mice lived for 25 days and mice in TK-deleted groups survived extended up to 36 days.

**Figure 6:**
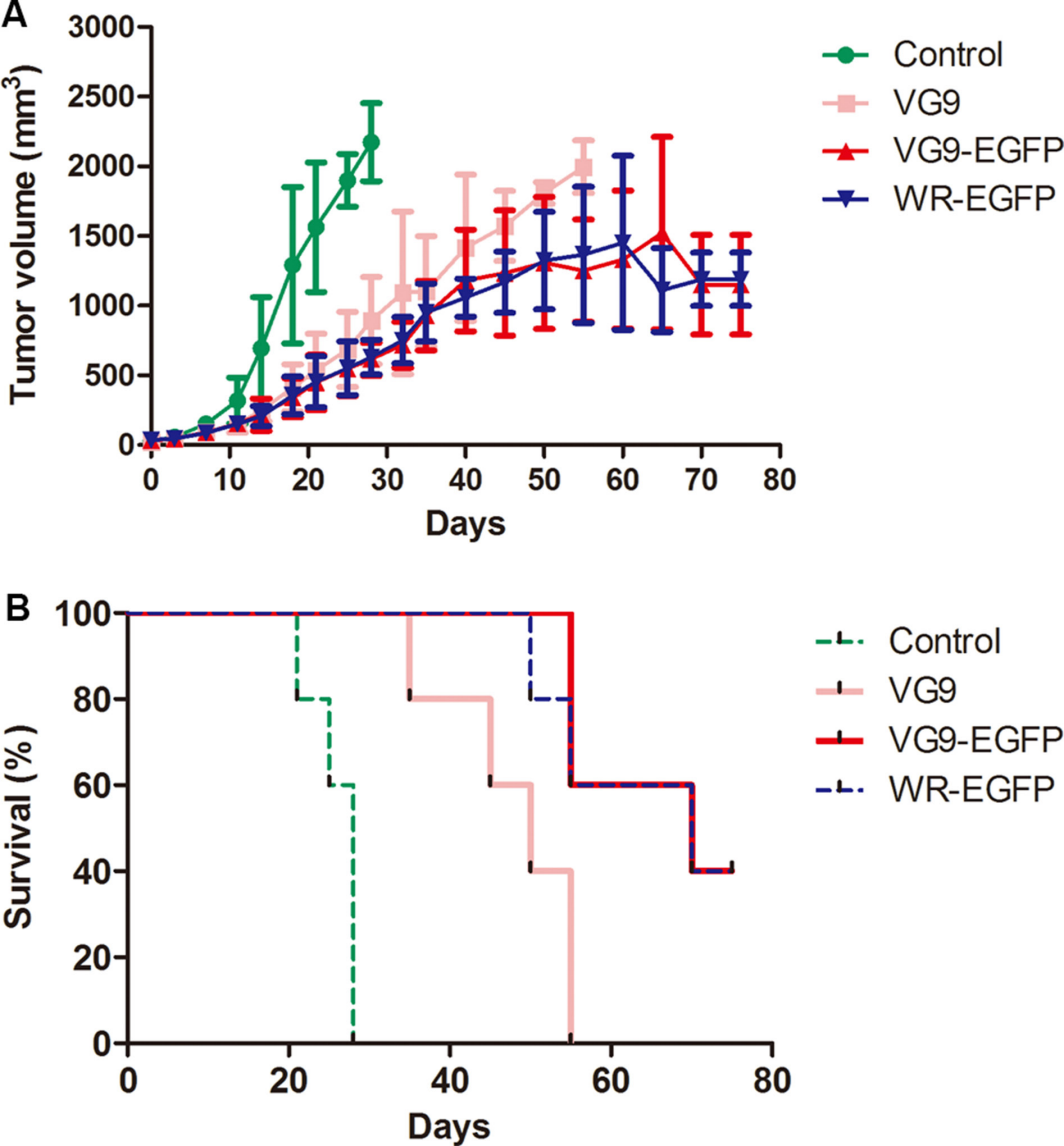
Antitumor efficacy in a human hepatoma tumor model (**A**) Mean tumor volume in mice treated with PBS (Control group), VG9 (VG9 group), or TK-deleted vaccinia virus (VG9-EGFP group, WR-EGFP group). (**B**) Kaplan-Meier survival curves for tumor-bearing mice treated with PBS (Control group), VG9 (VG9 group), or TK-deleted vaccinia virus (VG9-EGFP group, WR-EGFP group). *n* = 5 per group.

In the second human hepatoma tumor model, 6 × 10^6^ SMMC-7721 cells were injected subcutaneously into the left armpit of nude mice. 10 days after inoculation, PBS (control), VG9 or TK-deleted vaccinia virus (1 × 10^7^ PFU) was injected intratumorally. As is obvious in Figure 6, antitumor effect was significant in virus-treated mice (*P* < 0.01 as compared with control group). VG9-treated mice developed tumor and died with 55 days, while TK-deleted groups (VG9-EGFP and WR-EGFP) displayed prolonged survival. The antitumor effect of TK-deleted VG9 strain is as potent as TK-deleted WR strain. However, skin ulceration was observed in WR-EGFP treated mice, which was possibly due to higher virulence of WR strain.

Notably, the significant antitumor effect is due to the viral replication alone with no therapeutic gene insertion. All these results suggest that the TK-deleted vaccinia displays better antitumor efficacy.

## DISCUSSION

The generation of a recombinant vaccinia virus vector that specifically target and efficiently infect tumor tissue *in vivo* offers a potent approach to tumor-directed gene therapy. Such tumor-selective vector targeting and replication could theoretically cause direct lysis of tumor cells as well as enhance local therapeutic gene expression, leading to a significant antitumor effect.

Currently available vectors for tumor-directed gene therapy are nonviral delivery systems or replication defective viruses, such as retroviruses and adenoviruses. The inefficiency of these vectors limited their potential therapeutic effects. Herpes simplex virus has recently been investigated as a constitutive replication-competent virus in cancer treatment [33–35]. It is characterized similarly to vaccinia virus; however, the disadvantage of being a human pathogen limited its application. On the other hand, vaccinia virus has many attractive advantages over these other systems for cancer therapy. It is a large, cytoplasmic virus, with the capacity to accommodate multiple foreign genes. Unlike other viruses, it is independent on the cellular factors and carries its native and synthetic promoters to achieve high levels of transgene expression. Here, we have shown that vaccinia strain VG9 efficiently infects and expresses the EGFP marker gene in various cancer cell lines across different species. The most appealing feature, however, is the targeted nature and ability to replicate and express genes in tumor tissues. When we tested *in vivo*, VG9 specifically expresses the EGFP marker gene in tumor tissue compared with normal tissues. Lastly, there is a long history of human use with vaccinia virus clinically as a vaccine for the eradication of smallpox. The safety of vaccinia viruses in humans has been proven and specific antiviral agents are available [36, 37]. The predominant toxicity is mild flu-like symptoms, no treatment-related changes in the parameters of hematological, hepatic, and renal function and no significant normal tissue toxicity has been reported to date [38–40]. According to these favorable attributes, vaccinia virus has potential ability to be a novel platform for cancer gene therapy.

Nowadays, the complete mechanism of selective vaccinia infection of tumor cells has not yet to be fully elucidated. Several possible explanations are proposed. Vaccinia virus is known to replicate preferentially in metabolically active cells such like tumor cells, in which functional nucleotides are higher. Vaccinia virus encodes a thymidine kinase gene (TK) that, when deleted, leads to dependence of the virus on cellular thymidine kinase expression. Cellular thymidine kinase, which is regulated by the E2f transcription factors, is transiently expressed during the S phase of the cell cycle in proliferating normal cells, but is constitutively expressed at high levels in the majority of cancers regardless of proliferation status [41]. Therefore, TK-deleted vaccinia virus is dependent on host cell nucleotides, which compensate for the loss of viral TK, thus leading to predominant replication in tumor cells. Besides, as macromolecule, vaccinia virus is much easier to extravasate through permeable tumor vasculature than normal blood vessels [20]. Recent reports also indicate that the major factor in viral infection is the presence of heparin sulfate proteoglycans in the binding of the viral protein to cell surfaces [42]. Some evidences have suggested that heparin sulfate is more accessible within aggressively growing tumors [43]. Other studies revealed that vaccinia virus is inherently tumour-selective due to its dependency on the endothelial growth factor receptor (EGFR)-ras pathway [44, 45] as well as resistance to the type-I IFN response pathway [46, 47]. Future studies to illuminate mechanism clearly will make better application in cancer therapy.

In this study, we have shown that the TK-deleted vaccinia virus strain VG9 has significant antitumor effect both *in vitro* in various cancer cell lines and *in vivo* in murine or human tumor models. Such antitumor effects are as potent as TK-deleted WR strain, which has been widely used in laboratories and extensively tested in clinical trials. However, the virulence of WR strain is about 5000-fold higher than Chinese vaccinia strain Tian Tan (VTT) based on previous reports [48], while the virulence of VG9 in various animal models was found to be lower than its parental virus (VTT) [49]. TK-deletion can further reduce the virulence as well as enhance tumor targeting. Taking together, we conclude that TK-deleted VG9 may become a safer tumor-directed vector for cancer gene therapy.

In summary, we have shown here that TK-deleted vaccinia alone, without the presence of a therapeutic gene, is able to induce a notable antitumor effect through viral replication and subsequent cell death. In future, the antitumor effect may be enhanced by expressing suicide or immune cytokine genes as well as tumor-associated antigens or enzyme-prodrug. Given the enhanced oncolytic efficacy, tumor selectivity, and demonstrated safety, it is considered to be a promising therapeutic strategy in cancer treatment.

## MATERIALS AND METHODS

### Cell lines

Cell lines including HEK-293 (human embryonic kidney cell line), Vero (African green monkey kidney epithelial cell line), BSC-40 (African green monkey kidney epithelial cell line), B16 (murine melanoma cell line), Hepa1-6 (murine hepatoma cell line), A594 (human lung carcinoma cell line), MDA-MB-231 (human breast carcinoma cell line), SCG-7901 (human gastric carcinoma cell line), SMMC-7721 (human hepatocellular carcinoma cell line), L-O2 cells (human normal liver cell line), and NIH3T3 (murine embryo fibroblast cell line) were cultured under the conditions suggested by the American Type Culture Collection (ATCC, Manassas, VA, USA).

### Construction of TK gene deleted vaccinia virus

The vaccinia virus VG9 strain was obtained from National Institutes for Food and Drug Control (NIFDC, Beijing 100050, China). The vaccinia virus Western Reserve strain (WR) was kindly provided by Professor Liu (Institute of Biochemistry and Cell Biology, Shanghai Institutes for Biological Sciences, The Graduate School, Chinese Academy of Sciences). Enhanced green fluorescent protein (EGFP; GenBank: BAP87017.1) gene was inserted into TK locus to generate recombinant vaccinia virus. The vaccinia shuttle plasmid (pCB) used for construction was also kindly gifted from Professor Liu. The pCB plasmid is flanked by portions of the vaccinia TK gene (vTK-L, vTK-R), which facilitates homologous recombination into this locus (Figure 1A). The EGFP gene was under the control of the vaccinia synthetic early/late promoter [50] by cloning into the *EcoRI* and *XbaI* sites of pCB. After 2 hours infection with wild-type VG9, this recombinant shuttle plasmid was then transfected into HEK-293 cells by Effectene^®^ Transfection Reagent (Qiagen, Venlo, Netherlands). Recombinant isolation was performed in Vero cells by xanthine-guanine phosphoribosyltransferase (XGPRT) selection due to the presence of E.*coli gpt* gene. Following several rounds of selection, the virus was plaqued to confirm that a pure recombinant stock had been obtained. Vaccinia viruses were amplified in Vero cells and purified over a sucrose gradient centrifugation. Plaque-forming unit (PFU) virus titers were determined by plaque assay. The presence of recombinant vaccinia virus was observed by fluorescence microscope and verified by polymerase chain reaction (PCR). Total fluorescence was assayed at 488/597 nm with a spectrophotometric system (SpectraMax M5e, Molecular Devices, USA).

### Polymerase chain reaction

Viral DNA was extracted by Generay kit (Shanghai Generay Biotech Co., Ltd). A standard PCR was performed using primers external to the site of recombination (P1: 5′- ATGAACGGCGGACATATTCA-3′; P2: 5′-TTATGA GTCGATGTAACACTTTC -3′) and within the EGFP gene (P3: 5′-ATGGTGAGCAAGGGCGAGG-3′; P4: 5′-TTAC TTGTACAGCTCTCCATG-3′). The amplification was performed in 25-μl PCR reactions, containing 1× TaKaRa Taq buffer (MgCl_2_^+^), 1 U of Taq DNA polymerase, 0.2 mM of each deoxynucleoside triphosphate (dNTP), 0.04 μM of each primer, and 50 ng of viral DNA. PCR parameters consisted of 30 s denaturing (94°C), 30 s annealing (55°C), and 30 s or 2 min extension (72°C) for 35 cycles (2720 Thermal Cycler, Applied Biosystems, Waltham, MA, USA).

### Viral replication *in vitro*

Actively growing cells were prepared in advance, when dividing cells were become a confluent monolayer in 12-well plate, viruses were infected at the multiplicity of infection (MOI) of 0.1 PFU/cell. After incubated in growth medium containing 2% fetal bovine serum for 2 hours, cells were then incubated in complete growth medium. Cells and supernatant were harvested at indicated time points (24, 48, and 72 h). To establish resting cells, confluent NIH3T3 cells were incubated for 3 days in growth medium with 5% FBS and then were infected as above and harvested at the same time points. Viral titers were determined on BSC-40 cells after three cycles of freezing and thawing.

### *In vitro* cytotoxicity assay

Cells (10^4^/well) seeded in 96-well plates were infected with different concentrations of virus suspended in growth medium containing 2% fetal calf serum. Three days after infection, cell viability was analyzed by the MTT [3-(4, 5-dimethyl-2-thiazolyl)-2, 5-diphenyl-2-H-tetrazolium bromide] cytotoxicity assay.

### Mice

All animal studies were approved by the Institutional Animal Care and Use Committees (IACUC) of Jiangsu Institute of Nuclear Medicine (JSINM2010007). Female nude mice (7-week-old) and C57BL/6 immunocompetent mice (6-week-old) were purchased from Shanghai Laboratory Animals Center (SLAC; Shanghai, China). They were housed under standardized conditions with controlled temperature and humidity and a 12 h-12 h day-night light cycle. Mice were given free access to diet and water.

### Virus replication *in vivo*

To assess virus replication *in vivo*, C57BL/6 immunocompetent mice were intraperitoneally injected with VG9 or VG9-EGFP (1 × 10^7^ PFU). After 5 days, whole sections of brain, lung, liver, spleen, kidney, and tumor were harvested and homogenized, and a standard plaque assay was performed on BSC-40 cells.

### Tumor models and antitumor effects

To establish murine melanoma tumor model, about 5 × 10^5^ B16 cells in 100 μl phosphate-buffered saline (PBS) were injected subcutaneously into the right flanks of C57BL/6 mice. To establish human hepatoma tumor model, 6×10^6^ SMMC-7721 cells in 200 μl PBS were injected subcutaneously into the left armpit of nude mice.

When tumors reached the size of 3–5 mm in diameter, PBS (control), 10^7^ PFU of VG9, VG9-EGFP, WR-EGFP was injected intratumorally. Tumor growth was followed every other day and tumor volume was calculated as [(width)^2^ × length] × 0.52 [51]. Mice were euthanized when tumors reached their maximal permitted size according to the animal regulations, and Kaplan-Meier survival curves were plotted.

### Statistical analysis

Data are presented as mean ± standard deviation (SD). A statistical analysis was performed by using the Mann-Whitney test for nonparametric data or Student's *t*-test for two independent samples when appropriate. Survival analysis was performed using the method of Kaplan-Meier, and differences between curves were assessed using the log-rank test. All statistics were generated by SPSS 19.0 software (SPSS Statistics, Inc., Chicago, IL, USA).

## Abbreviations

TK: thymidine kinase
VG9: vaccinia strain Guang9
EGFP: enhanced green fluorescent protein
XGPRT: xanthine-guanine phosphoribosyltransferase
PFU: plaque-forming unit
MOI: multiplicity of infection
